# Visual Word Recognition in Deaf Readers: Lexicality Is Modulated by Communication Mode

**DOI:** 10.1371/journal.pone.0059080

**Published:** 2013-03-12

**Authors:** Laura Barca, Giovanni Pezzulo, Marianna Castrataro, Pasquale Rinaldi, Maria Cristina Caselli

**Affiliations:** 1 Institute of Cognitive Sciences and Technologies, National Research Council (ISTC-CNR), Rome, Italy; 2 Istituto di Linguistica Computazionale ‘Antonio Zampolli’, National Research Council (ILC-CNR), Pisa, Italy; University of Leicester, United Kingdom

## Abstract

Evidence indicates that adequate phonological abilities are necessary to develop proficient reading skills and that later in life *phonology* also has a role in the *covert visual word recognition* of expert readers. Impairments of acoustic perception, such as deafness, can lead to atypical phonological representations of written words and letters, which in turn can affect reading proficiency. Here, we report an experiment in which young adults with different levels of acoustic perception (i.e., hearing and deaf individuals) and different modes of communication (i.e., hearing individuals using spoken language, deaf individuals with a preference for sign language, and deaf individuals using the oral modality with less or no competence in sign language) performed a visual lexical decision task, which consisted of categorizing real words and consonant strings. The lexicality effect was restricted to deaf signers who responded faster to real words than consonant strings, showing over-reliance on whole word lexical processing of stimuli. No effect of stimulus type was found in deaf individuals using the oral modality or in hearing individuals. Thus, mode of communication modulates the lexicality effect. This suggests that learning a sign language during development shapes visuo-motor representations of words, which are tuned to the actions used to express them (phono-articulatory movements vs. hand movements) and to associated perceptions. As these visuo-motor representations are elicited during on-line linguistic processing and can overlap with the perceptual-motor processes required to execute the task, they can potentially produce interference or facilitation effects.

## Introduction

Theories of reading processes associate proficient reading skills with adequate phonological abilities because they mediate access to the meaning of written words, which is the basis of reading comprehension (for a review, see [1]). A specific dysfunction in learning to read (i.e., developmental dyslexia) has been recently associated with deficits in auditory temporal processing of language materials [2]. Strong evidence has been accumulated suggesting that *phonology* also plays a role in *covert visual word recognition*. Several brain imaging studies have suggested that reading a word covertly or deciding whether or not it is a real word involves a large network of cortical structures (usually located in the left hemisphere) including the occipito-temporal regions typically involved in visual recognition and the perisylvian speech network [3], [4]. The time course of the occipito-temporal and perisylvian regions indicates that phonological information does not strictly derive from previous recruitment of orthographic information but that there is much more on-line integration than previously suggested by the traditional sequential view of reading [5]. Thus, phonological processing seems to be automatically involved in processing written language, even when spoken output is not required.

One possible explanation is that covert phonological processes (inner speech) are adopted to support lexical processing. Consistent with this view, when reading a text we often have the subjective experience of inner speech, which has been shown to resemble our own voice [6]. Covert phonological processes (inner speech) might work as *emulators* of the to-be-recognized perceptual situation [7]–[9]; see also [10]. In this “emulation view”, predictive mechanisms (forward models) normally adopted for language production also support language perception by providing lexical (visual and/or auditory) predictions, similar to what forward models usually do in the context of motor control and the recognition of actions [11]. The emulation view predicts that difficulties in using phonological information should have detrimental effects on word recognition. A transcranial magnetic stimulation (TMS) study addressed this issue in the context of spoken language processing. The study showed that inducing interference by means of electrical stimulation at motor cortex sites controlling phonoarticulatory movements and, in particular, articulating the labials/p/and/b/and the palatals/d/and/t/affects auditory recognition of labial and palatal phonemes, respectively [12]. Consistent with the emulation view, the study suggests that phonological information (plausibly elicited through covert articulations) has a causal role in the auditory recognition of speech.

It still unclear, however, whether phonology plays a similar role in visual language processing and what happens when a complete and stable phonology is unavailable. Does the atypical processing of phonological information also impair reading processes? To answer this question, we investigated the visual word recognition skills of adult deaf readers. Impairments of acoustic perception might lead to atypical phonological representations of written words and letters based on letter-sound correspondence, which might affect deaf people’s proficiency in reading. The reduced or absent auditory input might produce a lack of phonological recoding/acoustic feedback, which is important in learning to read. At the same time, it should be noted that the development of phonological representations in deaf individuals does not necessarily depend on auditory speech experience, at either perceptual or production levels [13].

In the early stages of literacy acquisition, hearing children usually learn to read alphabetic scripts by creating a link between their spoken language lexicon and printed words together with a decoding strategy for translating letters to sounds, which allows reading unfamiliar words. Learning regular orthographies (i.e., Italian) is characterized by the transition from an initial stage of reading, based on phonological recoding, to a lexical stage, defined by analysis of the word as a whole [14]. The emergence of *lexical effects* (e.g., faster recognition of words frequently encountered in written text) in the early years of reading instruction, indicates that both reading procedures (letter-sound conversion and whole word recognition) are necessary and can work together to easily solve reading tasks [15], [16].

In the case of deaf children, a phonological recoding strategy might be more difficult to develop because their letter-sound knowledge is poor and might not provide an adequate basis for the development of a phonologically-based reading route. Beech and Harris [17] reported that hearing children rely on phonological coding to read English words more than deaf children. The fact that deaf children are not affected by the manipulation of spelling regularities supports the view that they mainly read via lexical activation using a sight-based vocabulary. This idea was challenged by Transler and Reitsman [18] who found that both hearing and deaf children made significantly more mistakes on pseudohomophones than control pseudo-words. Although pseudohomophone effects were smaller in deaf than hearing participants, the authors concluded that the deaf children also used phonological coding for written word recognition. Thus, although these studies report mixed results, there is some evidence for the involvement of phonology in reading in at least some deaf individuals. In the presence of unstable phonological representations, deaf readers might preferentially use a different reading strategy than hearing readers and might over-rely on whole-word lexical activations.

It is clear from this brief review that thus far the study of word recognition in deaf readers has produced inconsistent results. Miller [19], [20] reported that hearing Hebrew readers and oral deaf participants used the same phonologically-based strategy to solve a categorization task of visually presented words. By contrast, deaf signers have difficulty in tasks requiring phonological decoding abilities, but are as efficient as hearing people in recognizing and categorizing written words. This suggests that they have developed strategies for acquiring orthographic knowledge that do not rely on phonology. Deaf English readers, unlike hearing participants, showed no effect of word regularity [21] and were less affected by whether or not nonwords were pseudohomophones [22], [23]. This suggests that they rely more on whole word lexical processing than assembled phonology. In any case, methodological differences might explain the discrepant findings.

In studying language and cognitive functions in deaf individuals, several points need to be taken into account. The first point concerns the heterogeneity of the deaf population and the need for a critical distinction concerning the definition of *deafness*. There is great variability depending on the etiology of deafness (i.e., congenital or acquired), age of onset and diagnosis of hearing loss, degree of hearing loss, and whether the impairment involves one or both ears. Another key point regards the use of a cochlear implant. Different studies have pointed out that cochlear implantation can improve sound perception and phonological skills [24] even if it does not ensure the attainment of normal linguistic skills [25], [26]. Other related aspects concern the age when speech therapy started and the type of speech therapy (e.g. mainly oral communication or bilingual bimodal education) [27]. One of the most relevant variables is preferred *communication mode*, with a main distinction between individuals who prefer to use sign language (often defined as “deaf signers”) and those who prefer the oral modality and have less or no competence in a sign language (often defined as “oral deaf people”). Finally, deaf people can have access to some form of phonology through lip-reading or cued speech [28].

In the present study, young deaf adults who were readers of a shallow orthography performed a visual lexical decision task that involved categorizing words and consonant strings. When asked to read aloud, Italian readers are typically faster in naming words than nonwords regardless of the frequency of occurrence of the words (high or low), the list composition (pure vs. mixed blocks) [29], or the expertise of the readers (because the lexicality effect is present in adult expert readers as well as in beginning readers [30]). The use of consonant strings as non-lexical items is common in the neuroimaging literature on word recognition processes (see for example [31]–[33]), but less common in behavioral studies ([34]). Here, we chose to use consonant strings because they are non pronounceable, do not allow online building of a unitary speech-motor representation, and control better for the influence of phono-articulatory processes in written recognition than pronounceable words. Moreover, previous studies [17] have shown that legal nonwords are very difficult to process by deaf readers.

The purposes of the study were twofold: The first was to shed light on the written word recognition strategies used by deaf readers of a shallow orthography (Italian). We hypothesized that they would over-rely on a visual orthographic strategy to solve the task. The second was to test for an effect of communication mode by comparing the performance of deaf participants who preferentially use Italian Sign Language-LIS to communicate (here termed “*Deaf-LIS*”) and those who preferentially use Spoken Italian-SI (here termed “*Deaf-SI*”), and have less or no competence with LIS.

## Materials and Methods

### Ethics Statement

The procedure was approved by the Institute of Cognitive Sciences and Technologies of the National Research Council, ISTC-CNR of Rome. Informed consent was obtained from all participants and they were paid for participating.

### Participants

Thirty university students, aged 20–25 years, took part in the experiment. They were all right-handed according to the Edinburgh Handedness Inventory (laterality index >70%, [35]), with normal or corrected to normal vision. The deaf participants filled in an *Anamnestic Questionnaire.* This provided us with self-report information about years of education, experience with Italian Sign Language (LIS), frequency and context of LIS use, and family characteristics (e.g., deaf relatives), which we used to subdivide participants into groups.

#### Deaf with a preference for Italian Sign language (Deaf-LIS)

The group consisted of 10 deaf signers with severe to profound bilateral sensorineural hearing loss (71+ dB in the better ear). They had learned LIS in a family context (deaf children from deaf parents) or at school (deaf children from hearing parents), in a ‘naturalistic’ fashion, and within 3 years of age. They primarily use LIS for communication and adopt it in different social contexts (at home, at school, with friends). They also frequently use spoken language, mainly accompanied by corresponding signs. They attended mainstream schools with a ‘communication assistant’ who used LIS to communicate and to convey school subjects. Two participants regularly used hearing aids (none had a cochlear implant) when data were collected.

#### Deaf with a preference for Spoken Italian(Deaf-SI)

The group consisted of 10 deaf participants with severe to profound bilateral sensorineural hearing loss (71+ dB in the better ear). All of them were born from hearing parents and primarily used spoken language to communicate. They made limited or no use of LIS. Those who know LIS had learned it after 15 years of age. They all use hearing aids regularly and none had a cochlear implant when data were collected. They had attended mainstream schools with teachers who used spoken Italian to communicate and to convey school subjects.

Both groups underwent speech therapy during their school years.

#### Hearing Participants (HP)

The group consisted of 10 hearing participants who were monolingual, native speakers of Italian without any knowledge of sign language.

### Neuropsychological Assessment

Standardized tests were administered to ensure that participants had comparable cognitive, language and reading abilities. The first criterion for inclusion in the study was average or above average non-verbal intelligence as measured by a culture-free intelligence test, that is, the SPM (Raven’s Standard Progressive Matrices, [36]).

The second criterion was comparable reading skills between groups. For this purpose, we used an Italian reading comprehension test [37]. Specifically, participants were asked to read a story and answer 10 multiple-choice questions regarding its contents.

To measure vocabulary size, two fluency tasks were used. In the phonological fluency task (FAS), participants had to name as many words as they could beginning with the letters F, A and S in one minute. In the semantic fluency task (CAT), participants had to name as many items as they could belonging to a given semantic category (i.e., animals, foods, toys and jobs). In the semantic fluency task, both groups of deaf participants were asked to respond with spoken language in two categories and to use sign language in the other two (here we considered the overall number of correct responses produced, regardless of the modality). Participants’ scores are summarized in Table 1.

**Table 1:** Neuropsychological assessment.

	Chronological Age	SPM	Reading comprehension	FAS	CAT	Laterality index
Hearing*	22 (20–25)	119	72%	44	64	85
Deaf-SI	22 (20–23)	114	80%	31	50	98
Deaf-LIS	23 (20–24)	119	80%	24	60	95

Note. Chronological Age: mean age in years (range in brackets); SPM, mean test score; Reading comprehension: percentage correct responses; FAS: average number of words correctly produced in phonological fluency task; CAT: average number of words correctly produced in semantic fluency task; Laterality Index: average dexterity score.

* Values from 9 subjects only.

### Stimuli and Task

A list of lexical and non-lexical items was used. Lexical items were 100 Italian singular nouns taken from Barca, Burani and Arduino’s [38] database. All words were five letters long, morphologically simple (i.e., neither derived nor compounds), and unambiguous as to grammatical category and meaning. Words were acquired by age six and varied as to written frequency, that is, there were highly frequent words (e.g.,/MONDO/, ‘world’, with a frequency value = 2221) and less frequent items (e.g.,/ZUPPA/, ‘soup’, with a frequency value = 16). Written frequency is a measure of “adult written word frequency” taken from a frequency count based on a written corpus comprising 3,798,275 lexical occurrences (http://www.istc.cnr.it/material/database/colfis/indexeng.shtml). Overall, the list of words had a mean written frequency of 217±355. Words were all regularly stressed on the penultimate syllable.

Non-lexical items consisted of 100 strings of consonants. Letter strings were created by randomly assembling the consonants of the Italian alphabet. The letters ‘w’ and ‘y’ were not used. We chose consonant strings because they do not have word-like phonological or semantic representations and cannot be assembled in an articulatory manner (e.g.,/CPRTF/). Moreover, the use of pseudowords might have had a detrimental effect on deaf readers’ performance because legal nonwords (which resemble real words) are very difficult to categorize due to the transparency of Italian orthography and thus lead to a high number of errors ([34]; for deaf studies see [17]).

All participants were tested individually. Instructions were administered to deaf participants in their preferred communication mode by a hearing research assistant, who was an interpreter of LIS. Participants were asked to press (as fast and accurately as possible) a key on the computer keyboard if a word was presented (e.g., 1), and another key if a consonant string was presented (e.g., 0). The correspondence between stimulus type and button was varied across participants. Categorization errors and reaction times were automatically recorded by E-Prime software. Each trial began with a fixation cross in the center of the screen that was replaced by an experimental stimulus after 400 ms. Stimuli remained on the screen for 1000 ms. Participants had to respond within 1600 ms, otherwise a/TIME OUT/message appeared. Stimuli were presented in ARIAL font, upper case black print on a white background. The use of upper case letters allowed controlling for variations in the visual features of letters and words and ensured that the letters in the stimuli were always equally spaced and that the stimuli were the same length). Before the experimental data were acquired, participants performed a practice session with 10 non-experimental items (5 lexical and 5 non lexical stimuli). The experimental stimuli were presented in two blocks of 50 items each. The order of stimuli within blocks and the order of block presentation were randomized. The experimental session, including questionnaire and test administration, lasted about 1.5 hours.

## Results

Analyses were run on the data of 29 participants (the data of one Hearing participant were lost). Raw data are provided as Supporting Information. Overall, accuracy was very high (in the range of 94% to 96% in all conditions) and participants responded approximately 500 msec after stimulus presentation. Deaf-LIS responded incorrectly in 5.5% of cases (5.3% on words and 5.7% on consonant strings), Deaf-SI in 4.2% of cases (3.8% on words, 4.6% on consonant strings), and Hearing participants in 4.17% of cases (3.4% on words, 4.9% on consonant strings).

**Figure 1:**
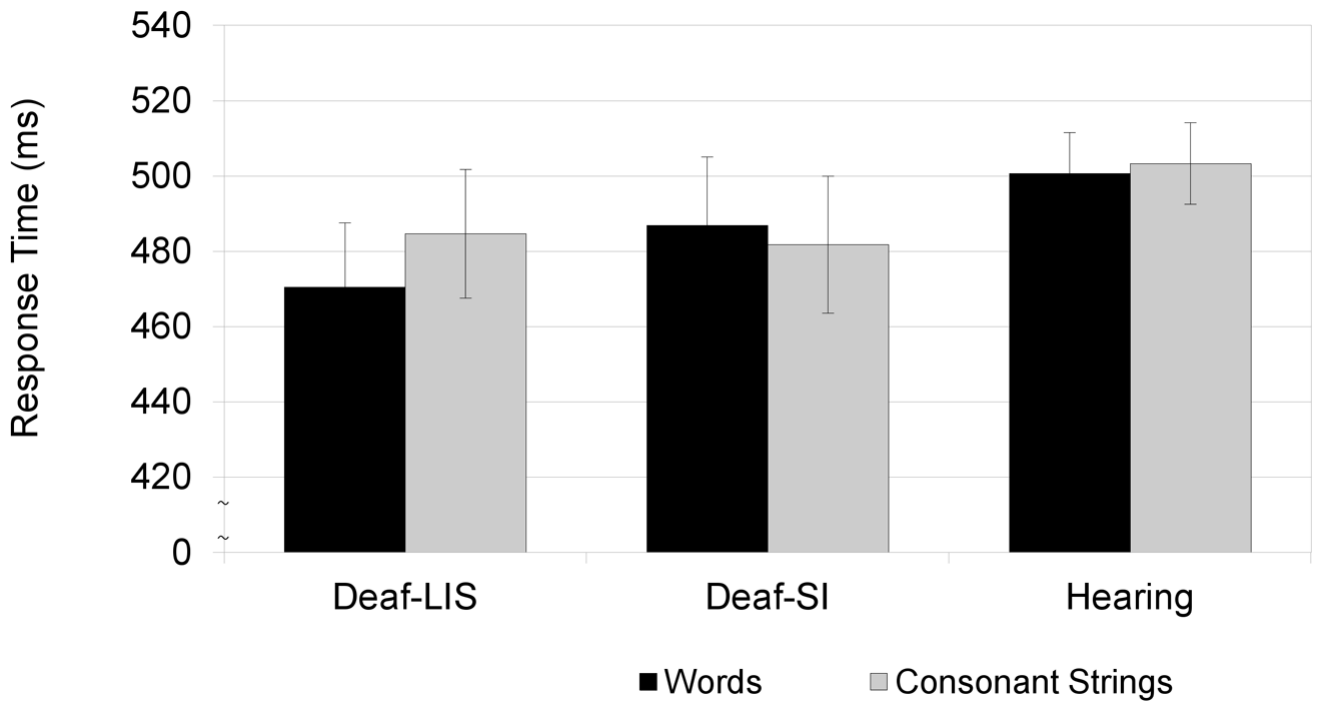
Visual Lexical Decision Reaction times. SEM in brackets.

Figure 1 displays the Group by Stimulus interaction, in which the lexicality effect is present only in the Deaf-LIS group. Performance of the Deaf-SI group was similar to that of the Hearing participants, with a lack of mean RT difference between words and consonant strings.

Correlations between RTs and percentage of correct responses performed separately for words and consonant strings in each group provided low correlation coefficients (ps >.1). The absence of a relationship between accuracy and speed undermines the possibility of a speed-accuracy trade-off.

### Statistical Analysis: Linear Mixed-Effects Modeling

Lexical decision data were analyzed by fitting the Linear Mixed-Effects Model (LMMs) to response time of correct decisions [39]. Logistic LMMs were used to analyze accuracy data. Analyses were run with the lm4 package for R [40].

#### Group effects on response times to words and consonant strings

To test for an effect of communication mode on response times, a mixed-effects analysis was performed separately for Words and Consonant Strings by comparing the responses of the three groups of participants. Subjects and Items were Random-effects factors and the Group category was the Fixed-effects factor (with performance of Deaf-LIS as the reference group). An absolute t-value exceeding 2 was considered to indicate significance difference (see [39]). On Word stimuli, RTs of Deaf-SI and Hearing did not differ from those of Deaf-LIS (β_Intercept_ = 484, tvalue = 25; β_Deaf-SI_ = 5.5, tvalue <1; β_Hearing_ = 15.7, tvalue <1), and no difference emerged from a direct comparison between Hearing and Deaf-SI (β_Intercept_ = 490, tvalue = 38.9; β_Hearing_ = 10.3, tvalue <1). Similar results emerged for Consonant Strings, with no difference between Deaf-SI and Hearing with respect to Deaf-LIS (β_Intercept_ = 500, tvalue = 24.4; β_Deaf-SI_ = −17.9, tvalue <1; β_Hearing_ = 3.2, tvalue <1) nor between Deaf-SI and Hearing (β_Intercept_ = 482.1, tvalue = 49.7; β_Hearing_ = 21.2, tvalue = 1). Thus, no reaction time differences emerged from a direct comparison of group performances on lexical and nonlexical stimuli.

To further investigate this lack of significant differences, we took into account individual performance on word-nonword categorization. Considering within-group variability, most Deaf-LIS (70%) showed the typical pattern, that is, they were faster in responding to words; 3 participants showed no effect (with less than a 12 msec difference). Differently, only 20% of a the Deaf-SI were faster in responding to words. In fact, most showed either no effects (60%) or the opposite pattern (20%), and one participant’s mean response time to consonant strings was 35 ms faster than to words. Finally, 33% of Hearing were faster on Words, 33% on Consonants strings, and the remaining participants in this group showed no difference. This suggests that there were individual differences within groups, but the small sample size prevented us from making further comparisons.

Considering response accuracy, no effects were significant on lexical or) nonlexical stimuli (Words: β_Intercept_ = 3.4, zvalue = 10.8, p<.001; β_Deaf-SI_ = .56, zvalue = 1.2, p>.1; β_Hearing_ = .36, zvalue = .8, p>.1; Consonant Strings: β_Intercept_ = 3.3, zvalue = 12.2, p<.001; β_Deaf-SI_ = .44, zvalue = .9, p>.1; β_Hearing_ = .31, zvalue = .6, p>.1 ).

#### Effects of lexicality on group response times

To test for an effect of stimulus lexicality within each group, separate LMMs analyses were performed with Lexicality as Fixed-factor and Subject and Items as random factors (with Consonant Strings as the reference level). A significant difference emerged for Deaf-LIS only. They responded faster to Words than Consonant Strings (β_Intercept_ = 500, tvalue = 24.5; β_Words_ = −15.3, tvalue = −2.5). No lexicality effects emerged for Deaf-SI (β_Intercept_ = 482, tvalue = 49.8; β_Words_ = 8.2, tvalue = 1.2) or Hearing participants (β_Intercept_ = 503, tvalue = 27.4; β_Words_ = −2.7, tvalue <1).

No significant effects emerged for accuracy: Hearing (β_Intercept_ = 3.6, zvalue = 8.1, p<.001; β_Words_ = .15, zvalue = .49, p>.1), Deaf-LIS (β_Intercept_ = 3.7, zvalue = 11.3, p<.001; β_Words_ = −.25, zvalue = −.83, p>.1), and Deaf-SI (β_Intercept_ = 3.6, zvalue = 10.1, p<.001; β_Words_ = .32, zvalue = 1.2, p>.1).

#### Lexicality and language fluency tasks

A correlational analysis of the lexicality scores (i.e. [RT words – RT consonant strings]) and the neuropsychological scores of each group revealed a moderate relationship between lexicality and FAS value for Deaf-LIS only (r_Deaf-LIS_ −.54, p = .068).

**Figure 2:**
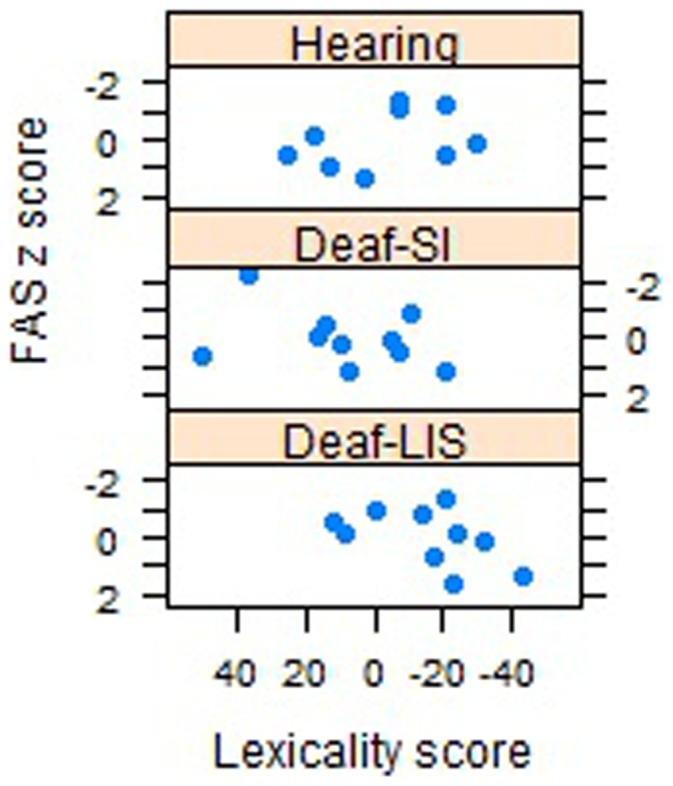
Lexicality effect and phonological fluency task. Scatterplot depicting the mean number of correct responses for each participant within groups (FAS z score), plotted against reaction time difference score between words and consonant strings (Lexicality score).

**Figure 3:**
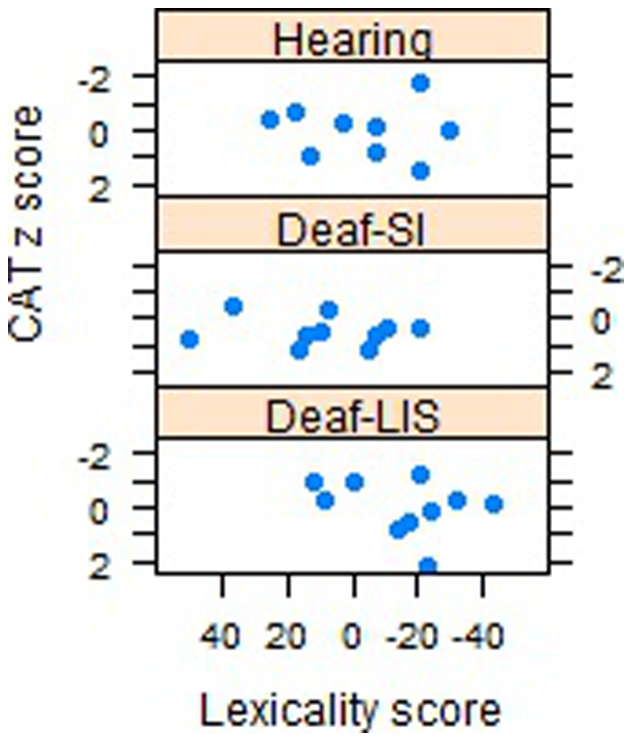
Lexicality effect and semantic fluency task. Scatterplot depicting the mean number of correct responses for each participant within groups (CAT z score), plotted against reaction time difference score between words and consonant strings (Lexicality score).

Figures 2 and 3 illustrate the relationship between lexicality scores and vocabulary fluency tasks. Participants with a lexicality score equal (or close) to zero showed no reaction time difference between words and consonant strings; negative values indicate faster response to words, positive values indicate faster response to consonant strings. The direction of the correlation indicates that participants who showed a greater difference between words and consonant strings had a richer vocabulary (i.e., they produced more words in the fluency tasks). No other correlations were significant. As noted above, the lexicality pattern was less clear for Hearing and Deaf-SI than for Deaf-LIS. Deaf-SI showed much more variability; in fact, two participants clearly showed the reversed lexicality effect (Participants 18 and 19).

## Discussion

There are many published studies on cognition in the deaf that report divergent results including no change, enhanced or even worse performance (compared with hearing participants) on a variety of linguistic tasks. This lack of agreement could be due to methodological issues, because the deaf population is very heterogeneous. Factors such as age of diagnosis, degree of hearing impairment, age at first exposure to sign language and preferred communication modality (i.e., sign based or oral) have to be taken into account [28], especially when considering the impact of auditory deprivation on literacy skills. Here we controlled for all of these factors by comparing written language processing in participants who communicate with Spoken Italian or primarily Italian Sign Language. Deaf participants were either deaf individuals who communicate mainly with sign language, which they learned ‘naturally’ at home before age 3, or deaf individuals who prefer spoken language (learned via formal instruction) and have poor fluency in sign language. Hearing participants with knowledge of spoken language but not sign language constituted the reference group. The three groups were similar for chronological age, reading and language experience and educational level. The visual lexical decision paradigm is typically used to evaluate ease of access and retrieval of lexical information stored in memory, with real words recognized faster and more accurately than nonwords. Results suggest that different recognition strategies might be in play when deaf individuals and hearing participants categorize legal Italian words and illegal letter strings.

The key finding of the study is that the *Lexicality Effect* was present only in Deaf-LIS; in fact, this group categorized the typical pattern of lexical items faster than the non lexical items. No such difference emerged for Deaf-SI or Hearing participants. Generally, the time needed to make a decision decreased because we used consonant strings as nonlexical contrast. This finding is different from those of studies that used legal pseudowords [41], because stimulus discrimination does not require in-depth analysis but can be based on visual processing of items, resulting in overall faster decision times. This might also explain the lack of a lexicality effect in the hearing participants. In fact, it suggests that they mainly use a visual strategy based on the physical features of the stimuli. The absence of a response time difference between words and letter strings was recently reported in a kinematic study of the visual lexical decision paradigm [34]. The use of consonant strings probably affected the strategy used by participants to solve the task. Indeed, an important topic for future research would be to assess whether using more common nonlexical stimuli would produce different results.

A popular view is that deaf individuals have enhanced vision to compensate for auditory deficits, and indeed a number of studies report increased processing speed of deaf participants on several tasks, due to faster reactions to visual stimuli and enhanced visual attention [42], [43]. This evidence has been used to support the *sensory compensation hypothesis,* according to which deaf individuals develop better visual functions to compensate for their lack of stable auditory input. Nevertheless, sensory compensation does not hold for the entire visual cognition domain but is rather specific to its sub-components [44], particularly the processing of stimuli located in the visual periphery, stimuli in motion, and in conditions requiring attention selection, that is, all conditions not present in our study (see for a review [45]).

On a different ground, *processing written language* might be different in Deaf-LIS and reflect greater reliance on whole word visual processing and orthographic knowledge. The negative correlations between the lexicality effect and the fluency task support this reasoning. Results are consistent with several experimental paradigms showing that many deaf individuals rely on visual information when reading, encoding and processing written English [46], [47].

Results suggest that communication mode modulates cognitive processes because only Deaf-LIS were sensitive to the lexicality of the stimuli; in fact, Deaf-SI performed like Hearing participants. One possibility is that the increased speed of Deaf-LIS in categorizing real words over strings of consonants might be related to enhanced reliance on ‘whole word visual-orthographic processing’ as an outcome during literacy acquisition. In fact, from the first stages of formal instruction this is the primary modality through which this group learns literacy skills. Specifically, Deaf-LIS learn words as a whole rather than focusing on individual letters (i.e., the 'phonic' method usually adopted in transparent orthographies). In this vein, an enhanced visual strategy in processing written language might be the consequence of an increase in the allocation of attention resources to perceptual stages of the recognition process. The use of legal words and illegal letter strings probably enhanced this sight recognition.

One question remains regarding the locus of deaf signers’ performance in current word recognition models. According to the Dual-Route Cascaded model [48], a lexical route activating word units operates in parallel with a sublexical route in which the pronunciation of any letter string is accomplished through grapheme-to-phoneme conversion rules that apply in a serial, left-to-right fashion. In this model, differences might arise at an earlier stage than that of the two reading procedures, namely, at the level of visual analysis However, given that the visual level analysis is neutral regarding subsequent activation of the lexical or nonlexical reading procedure [49], the presence of the lexicality effect might lead to rejection of this locus. Nevertheless, direct manipulation of variables such as stimulus length or frequency of occurrence might provide compelling evidence against or in favor of this idea.

Another possibility is that deaf signers over-rely on lexical reading. Consistent with this, recent studies suggest that both orthographic and sign language lexicons are activated during written language processing [43]. Using sign language as a communication mode from infancy might shape motor and language neural circuits, resulting in richer visuo-motor representation of words in terms of a sign-based phonological representation, and enhancement of semantic activation of cerebral regions, such as the inferior parietal lobule (IPL), which are related to the coding of motor acts and praxis information in human [50], [51] and non-human primates [52]. In line with this reasoning, results might be interpreted within a framework that sees action and language networks as deeply connected [33], [53], [54]. This study adds to the picture the idea that language is tightly connected to the actions used to express it (for Deaf-LIS, mainly hand and upper limb movements, not phono-articulatory processes) and associated perceptions (for Deaf-LIS, mainly visuospatial recognition of gestures and speech reading). In keeping with this idea, Elliot et al. [55] proposed a model of single-word reading in deaf signers, adapted from the DRC model. In this model, deaf individuals are thought to have the same architecture as hearing individuals. The main difference is that the sublexical units are not grapheme-to-phonemes but grapheme-to-'visemes' (i.e., visual phonemes derivable from speechreading). Further empirical testing is needed to corroborate this interesting hypothesis, which might fit with our last argument.

Finally, Deaf-LIS might respond faster because their preferred communication mode (hand movements) is closely related to the task demands (i.e., responding with a hand movement). Models of spoken language postulate a functional link between motor and perceptual representations of speech, and there is mounting evidence that this link is causal in nature [12], [53]. In the case of deaf native signers, language production is based on motor system programming, and controlling and executing upper limb and hand movements, which are also in charge of programming, controlling and executing the motor component to perform the task (i.e., key pressing with the index finger of the left and the right hand). For Deaf-LIS, pre-activation of language-related hand movement circuits might lead to faster RTs for lexical items. This idea can be explained within the “emulation view” discussed in the Introduction. This view assumes that internal forward models are automatically activated during perceptual tasks to support perceptual processing (in this case, word recognition). As their communication mode is manual, Deaf-LIS might predominantly use forward models of arm movements. In turn, as forward models enact covert hand movements, they might elicit hand response codes and thus produce faster manual responses.

The results of our study do not allow us to judge between these competing hypotheses. In either case, however, our reasoning emphasizes the importance of communication modes in written language processing. During development, communication modes shape visuo-motor representations of words that are tuned to the actions used to express them and the associated perceptions. During on-line linguistic processing, visuo-motor representations are elicited and can overlap with the perceptual-motor processes required to execute the task, potentially producing interference or facilitation effects.

In conclusion, our results indicate that language modality affects written language processes also in shallow scripts (Italian). The present study showed that deafness does not necessarily cause individuals to fail in efficiently processing visually presented words. Given the transparency of Italian orthography, Italian deaf readers might have an edge with respect to deaf readers of other orthographies (see [19], [20]) because they can rely more on visual-orthographic word recognition to perform a visual lexicality decision task. The deaf readers who preferred to communicate with LIS were sensitive to stimulus lexicality, whereas the deaf readers who preferred Spoken Italian performed similarly to the hearing controls. Additional studies are needed to directly test the use of different reading strategies by deaf individuals (for example, using legal nonwords for comparison) and to determine whether and how deaf signers are sensitive to the lexical principles of written language.

## Supporting Information

Dataset S1 **(file format Excel 97/2000/XP) provides Lexical Decision response time for correct categorizations.**
(ZIP)
