# Supplementary material for: Visual Word Recognition in Deaf Readers: Lexicality Is Modulated by Communication Mode
Source: PLoS One. 2013 Mar 12;8(3):e59080. doi: 10.1371/journal.pone.0059080 (PMC3595400; doi:10.1371/journal.pone.0059080)
Supplement: Dataset S1 — (file format Excel 97/2000/XP) provides Lexical Decision response time for correct categorizations. (ZIP) [file pone.0059080.s001.zip › Legend S1 of Dataset S1.doc]

Supplementary material for the manuscript

**'Visual word recognition in deaf readers:**

**Lexicality is modulated by communication mode'**

Laura Barca^1^, Giovanni Pezzulo^1,2^, Marianna Castrataro^1^,

Pasquale Rinaldi^1^, Maria Cristina Caselli^1^.

^1^Institute of Cognitive Sciences and Technologies, National Research Council (ISTC-CNR), Rome, Italy

**^2^**Istituto di Linguistica Computazionale 'Antonio Zampolli', National Research Council (ILC-CNR), Pisa, Italy

The manuscript reports an experiment in which young adults with different levels of acoustic perception (i.e., hearing and deaf individuals) and different modes of communication (i.e., hearing individuals using spoken language, deaf individuals with a preference for sign language, and deaf individuals using the oral modality with less or no competence in sign language) performed a visual lexical decision task, which consisted of categorizing real words and consonant strings.

Supplementary Material.xls (file format Excel 97/2000/XP) provides Lexical Decision response time for correct categorizations.

Information provided are:

- Lexicality of the stimuli (Word/LetterString, column A),
- Stimuli presented (column B),
- identification number of each participant (row 2)
- Group: Deaf-LIS (deaf participants who preferentially use Italian Sign Language-LIS to communicate), Deaf-SP (deaf participants who preferentially use Spoken Italian-SI, and have less or no competence with LIS), Hearing (hearing participants who use spoken italian to communicate and have no competence with LIS).
